# Space–time LAI variability in Northern Puglia (Italy) from SPOT VGT data

**DOI:** 10.1007/s10661-015-4603-6

**Published:** 2015-06-16

**Authors:** Gabriella Balacco, Benedetto Figorito, Eufemia Tarantino, Andrea Gioia, Vito Iacobellis

**Affiliations:** Politecnico di Bari, via Orabona 4, Bari, 70125 Italy; ARPA PUGLIA, C.so Trieste 27, Bari, 70126 Italy

**Keywords:** Leaf area index, SPOT VGT, Time series analysis

## Abstract

The vegetation space–time variability during 1999–2010 in the North of the Apulian region (Southern Italy) was analysed using SPOT VEGETATION (VGT) sensor data. Three bands of VEGETATION (RED, NIR and SWIR) were used to implement the vegetation index named reduced simple ratio (RSR) to derive leaf area index (LAI). The monthly average LAI is an indicator of biomass and canopy cover, while the difference between the annual maximum and minimum LAI is an indicator of annual leaf turnover. The space–time distribution of LAI at the catchment scale was analysed over the examined period to detect the consistency of vegetation dynamics in the study area. A diffuse increase of LAI was observed in the examined years that cannot be directly explained only in terms of increasing water availability. Thus, in order to explain such a general behaviour in terms of climatic factors, the analysis was performed upon stratification of land cover classes, focusing on the most widespread species: forest and wheat. An interesting ascending–descending behaviour was observed in the relationship between inter-annual increments of maximum LAI and rainfall, and in particular, a strong negative correlation was found when the rainfall amount in January and February exceeded a critical threshold of about 100 mm.

## Introduction

The process of mapping, quantifying, and monitoring changes in the physical characteristics of vegetation cover has become essential to understand the dynamics of state variables in hydrologic, agricultural, and ecologic systems from regional to global scales (Miao et al. 2013; Nemani et al. 1996; Peng et al. 2012; Nolè et al. 2014). Trend analysis of remote-sensing time series has been used in many studies to effectively describe a vegetation trend in natural environments (Sonnenschein et al. 2011) and agricultural ecosystems (Tottrup and Rasmussen 2004; Dubovyk et al. 2012).

Thanks to their continuous recording, extensive coverage and cheaper costs in comparison with more recent very high resolution Earth Observing satellites, LANDSAT and SPOT series are still commonly used to study such endeavours (Kidane et al. 2012; Kovacs et al. 2009; Ekercin and Örmeci 2010; Röder et al. 2008; Yu et al. 2012). Long-term operational satellite missions have been providing an increasing amount of information, which requires advanced and effective methodological approaches to combine digital image-processing techniques with statistic time series analysis (Telesca et al. 2008; Telesca and Lasaponara 2008) in order to obtain information on features and causes of variations at different time scales (Telesca and Lasaponara 2005; Gessner et al. 2013; Atzberger and Eilers 2011).

Although the multi-temporal coarse resolution of satellite images clearly restricts the range of spatial details that can be detected, they have proved to be suitable for identifying locations of rapid change for further analysis with higher resolution data (Gutman and Masek 2012). However, extracting trends from time series can be challenging due to short-term (e.g. phenological) variations in the data or overall low signal-to-noise ratios (Verbesselt et al. 2010). It is hence necessary to establish long enough time series for a reliable critical historical perspective on vegetation activities (Brown et al. 2006; Qiu et al. 2013) to determine effective indicators of the degree of stress resulting from natural hazards and/or anthropogenic activities (Telesca et al. 2008; Lanorte et al. 2014; Vancutsem et al. 2009).

On the other hand, the detail and frequency of acquisition of multi-temporal coarse resolution satellite images seem suitable for their exploitation within hydrological models applied at basin scale. In a semi-arid Mediterranean environment, the hydrological water balance is highly conditioned by land cover and vegetation dynamics. For example, evapotranspiration, which is recognised as the main hydrologic loss (50 ÷ 60 % of mean annual rainfall, e.g. Maidment 1992), can be evaluated as the sum of two distinct processes: evaporation from bare soil and transpiration from vegetative soil. In drought conditions, the evaporation from bare soil may increase, limited by water availability, while canopy transpiration generally decreases. Therefore, the space–time distribution of vegetation is a key factor for a correct evaluation of evapotranspiration (Liu et al. 2009; Gigante et al. 2009) and also for understanding climate–soil–vegetation interactions (Portoghese et al. 2008).

The total leaf area quantity per unit area, namely the leaf area index (LAI), rapidly responds to different stress factors and changes in climatic conditions, proving very useful as a consistent indicator to characterise the vegetation condition commonly used in hydrologic and climate models (Fassnacht et al. 1997; Stenberg et al. 2004; Houborg and Boegh 2008; Zheng and Moskal 2009). LAI estimates from coarse resolution sensor data are needed by the scientific community investigating global scale fluxes and energy balance of the land surface because they provide an indication of vegetation growth cycle and, as such, of the plant activity in terms of water transpiration (Gigante et al. 2009). Measuring LAI is not a straightforward task, especially over a heterogeneous landscape (Martinez et al. 2010). LAI varies in space obviously depending on the vegetation type. LAI also varies for any fixed vegetation type in time and space depending on the stage of development and crop practices (Aquilino et al. 2014). Since direct measurements of LAI are usually time-consuming and require continuous updates, remote measurements from optical, SAR and LIDAR sensors were demonstrated to be an alternative to estimate this attribute both over large areas and at watershed scale (Alberti et al. 2013; Capodici et al. 2013; Piragnolo et al. 2014; Rinaldi et al. 2010). A number of global LAI products are being routinely estimated using data of spatial moderate and coarse resolution from various satellite sensors such as MODIS, SPOT and MERIS, having a frequency of 1–2 weeks and timely covering a period of somewhat more than 1 decade (Chen et al. 2006; Propastin and Kappas 2012). Validating these products is a difficult task because ground-based plot measurements are always limited and cannot be compared with these image data directly without considering surface heterogeneity. Major issues facing LAI product validation may include (1) consistency in ground-based LAI measurement methods and protocols since there have been different definitions of LAI and diverse methods of LAI estimation, (2) methods for spatial scaling from ground plot to pixel, and (3) accuracy assessment for coarse-resolution LAI images (Chen et al. 2002). For LAI estimation, some spectral vegetation indexes using the ratio of combined red and near-infrared (NIR) reflectance, such as the normalised difference vegetation index (NDVI) or the simple ratio (SR), are commonly used (Stenberg et al. 2004), even if some studies suggest that NDVI and LAI are strongly correlated (Andersen et al. 2002). Chen et al. (2002) made use of the reduced simple ratio (RSR) method based on a previous study documenting its ability in retrieving LAI index when dealing with heterogeneous land covers.

In this paper, time series SPOT VEGETATION (VGT) satellite archives were used for retrieving LAI on a study area located in the North of Apulia, a region in Southern Italy. The variability in space and time of such index at the catchment scale over the period 1999–2010 and its correlation with temperature and rainfall observations was analysed, in order to investigate the consistency of LAI variability and to characterise the vegetation dynamics to be developed in hydrologic and climate models.

## Site description

**Fig. 1:**
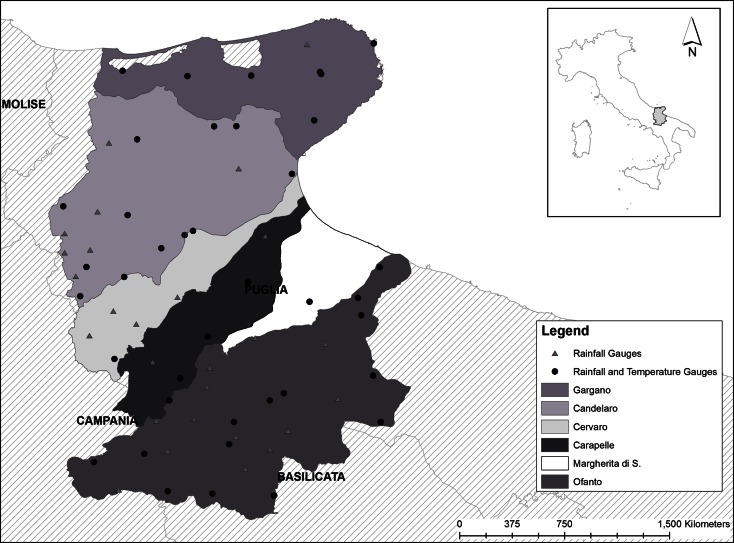
Study area in the North of Apulia (Italy)

The study area is in Apulia, the most Eastern region of Italy overlooking the Adriatic and Ionian seas, and it spreads across its Northern territory including the Gargano promontory, the Tavoliere plain, the river basins Candelaro, Cervaro, Carapelle, Ofanto and the reclamation ground of Margherita di Savoia (Fig. 1).

The Gargano promontory has a geological matrix that is exclusively limestone with high plains covered in forest. Land use is very complex, with many permanent meadows in the upper part, intermingled with forest, mainly high forests and coppices, which cover about 50,640 ha (Lovreglio et al. 2010).

The Apulian Tavoliere is the second largest Italian plain, and agriculture is its first productive activity. It includes arable lands producing cereals and vegetables, olives (including century-old tree specimen), fruit orchards and vineyards, together with spontaneous vegetation typical of Mediterranean Macchia. The spacing of trees is affected by local agricultural practices aimed at maximising productivity and reducing disease exposure. A marked differentiation exists between seasonal and permanent vegetation (for instance between winter wheat and olives). Seasonal wheat crops are usually characterised by an almost complete vegetation ground cover with a high root density and shallow root depth. Instead, permanent tree crops, mainly olives, grapes and citrus, have lower percentages of vegetation ground cover, deeper roots and lower root density.

The site is characterised by Mediterranean heterogeneous climate with strong inter-annual variability and a marked annual seasonality. Rainfall is distributed quite irregularly over the year with average minimum value of about 600 mm/year and peaks in the October–March semester.

## Data and methodology

The analysis was conducted on SPOT VGT time series images at a spatial resolution of 1 km at nadir, for the period 1999–2000. The VEGETATION (VGT) instrument encompasses four spectral bands in the blue, red, near-infrared and shortwave-infrared spectral wavelengths. The orbit of the VGT sensor ensures daily global coverage of the Earth’s surface with a 1-km footprint of the pixel in the ground. The Vlaamse Instelling voor Technologisch Onderzoek (VITO) routinely operates atmospheric and angular corrections of reflectance data from SPOT-4/VGT-I and SPOT-5/VGTII, which results in two 10-day composite products (S10 and D10 data) delivered in a Plate Carrée projection (WGS84 ellipsoid), at a spatial resolution of 1/112° (Maisongrande et al. 2004).

Climatic data on monthly rainfall and temperatures were provided by the Regional Agency for Hydrographic Monitoring (Servizio Idrografico—Regione Puglia) and related to 66 rainfall gauges and 39 temperature gauges, covering the period 1999–2000. Their localisation is shown in Fig. 1.

### SPOT VGT data processing

To facilitate the comparison of multi-temporal imageries, it was necessary to register the data set in a single map coordinate system, in this case Universal Transverse Mercator projection (zone 33) and datum WGS84.

Each composite product consists of two 8-bit data files: a digital number (DN) file to be converted into NDVI and a status map (SM) file describing some potential problems observed for the reflectance of each band during the period of compositing (Hagolle et al. 2005). Using 12 years (from January 1, 1999, to December 31, 2010) of VGT data, we used VGT-S10 products (10-day synthesis) that were compiled by merging segments (data strips) acquired over 10 days. These are mosaics of the acquired image segments, where each pixel contains the best possible observation during a 10-day period.

The VGT datasets were atmospherically corrected through the use of SMAC software (Rahman and Dedieu 1994; Passot 2000; Verbesselt et al., 2007), which is a simplified implementation of the 6S method (Vermote et al. 1997). The post-processing steps include reprojection and reflectance calibration. The raw VGT data are in Plate Carrée projection with a 0.0089285714° pixel resolution. In this study, data were transformed and resampled using a nearest neighbour operator into the UTM projection based on WGS84 spheroid at 1-km resolution within *VGTExtract* tool created by VITO. Then, the Savitzky–Golay filter was used to fill the gap (Chen et al. 2004; Atzberger and Eilers 2011), reported in the status map (SM) file. A status map indicating image quality, snow and ice, cloud and cloud shadows was also delivered.

Three bands of SPOT-VEGETATION (red, NIR and SWIR) were used in this study to form a vegetation index named reduced simple ratio (RSR) and defined as in (Brown et al. 2000)

1 $$ \mathrm{R}\mathrm{S}\mathrm{R}=\frac{\rho_{NIR}}{\rho_{RED}}\left(1-\frac{\rho_{SWIR}-{\rho}_{SWIR \min }}{\rho_{SWIR \max }-{\rho}_{SWIR \min }}\right) $$

where

- *ρ*_NIR_, *ρ*_RED_ and *ρ*_SWIR_ are the reflectance in NIR, RED and SWIR band, respectively.
- *ρ*_SWIRmin_ and *ρ*_SWIRmax_ are the minimum and maximum SWIR reflectance found in each image and defined as the 1 % minimum and maximum cut-off points in the histograms of SWIR reflectance in a wide scene.

In principle, the major advantages of RSR can be summarised as follows:

- The difference between cover types is very much reduced so that the accuracy for LAI retrieval for mixed cover types can be improved, or a single LAI algorithm can be developed without resorting to a coregistered land cover map as first approximation.
- The background (understory, moss cover, litter and soil) influence is suppressed using RSR because the SWIR band is most sensitive to the amount of vegetation containing liquid water in the background (Brown et al. 2000).

By using RSR, the background value was no longer needed. However, this does not mean that the background effect was completely removed. The extent to which the background effect is suppressed depends on how well the minimum SWIR represents reflectance for the whole scene. For LAI calculations of cropland, grassland, tundra, barren and urban, Chen et al. (2002) implemented the following algorithm based on RSR:

2 $$ \mathrm{L}\mathrm{A}\mathrm{I}=\mathrm{R}\mathrm{S}\mathrm{R}/1.3 $$

## Analysis of space–time patterns

**Fig. 2:**
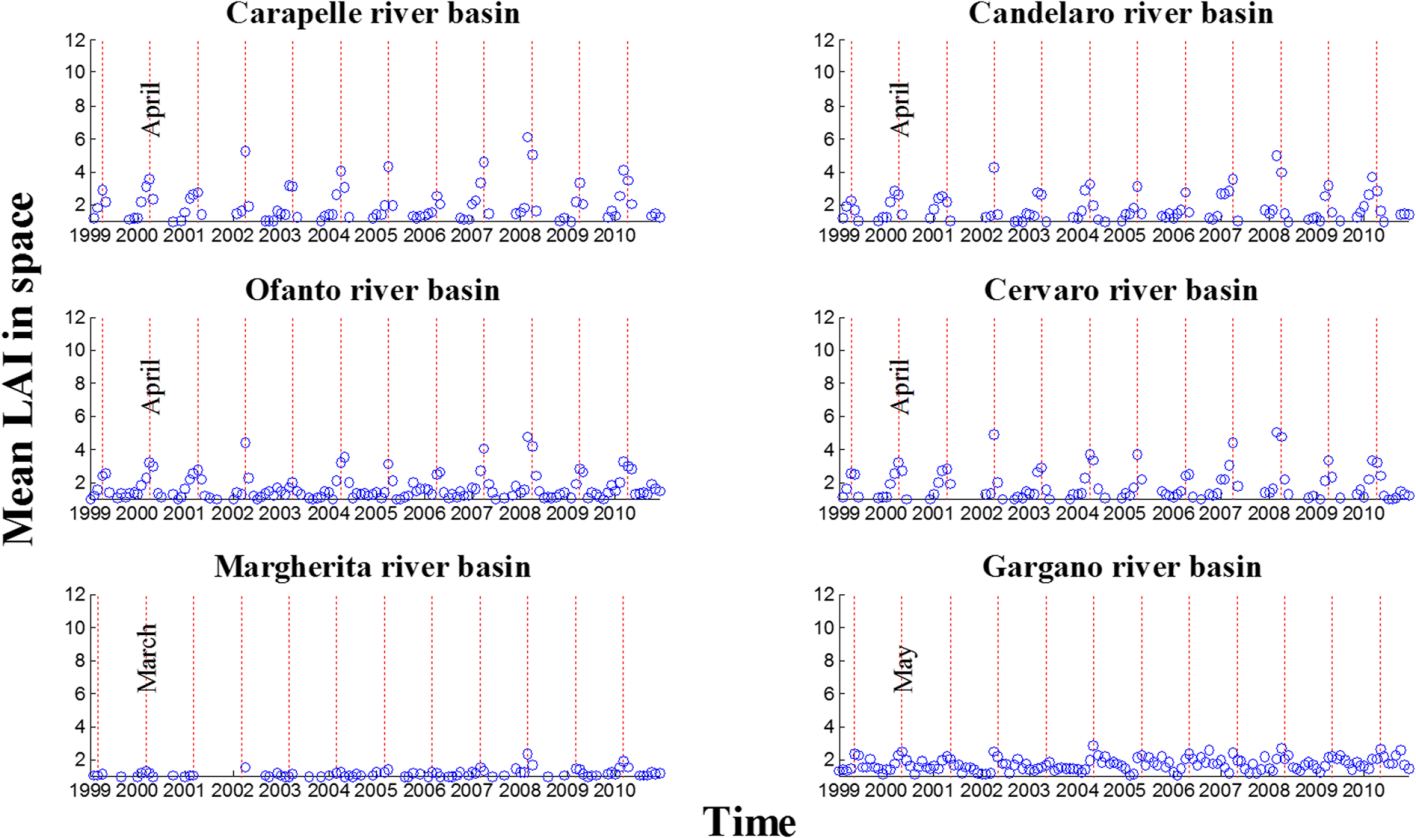
Time series of monthly LAI for the years 1999–2010

The extensive database obtained by means of SPOT VGT images allows for a comprehensive analysis of LAI variability in time and space. With this purpose, the first results are shown with reference to monthly time series of spatial-average LAI. The charts in Fig. 2 report these time series of monthly LAI. The blue circles represent the spatial average for each month in the period; the (vertical) red dashed lines indicate the month of April of each year when the LAI maximum value of wheat was observed. Consistently, the subplots relative to Candelaro, Carapelle, Cervaro and Ofanto, basins with a significative presence of wheat, showed their annual maximum LAI in April throughout the whole study period, with the only exceptions of years 2008 and 2010.

**Fig. 3:**
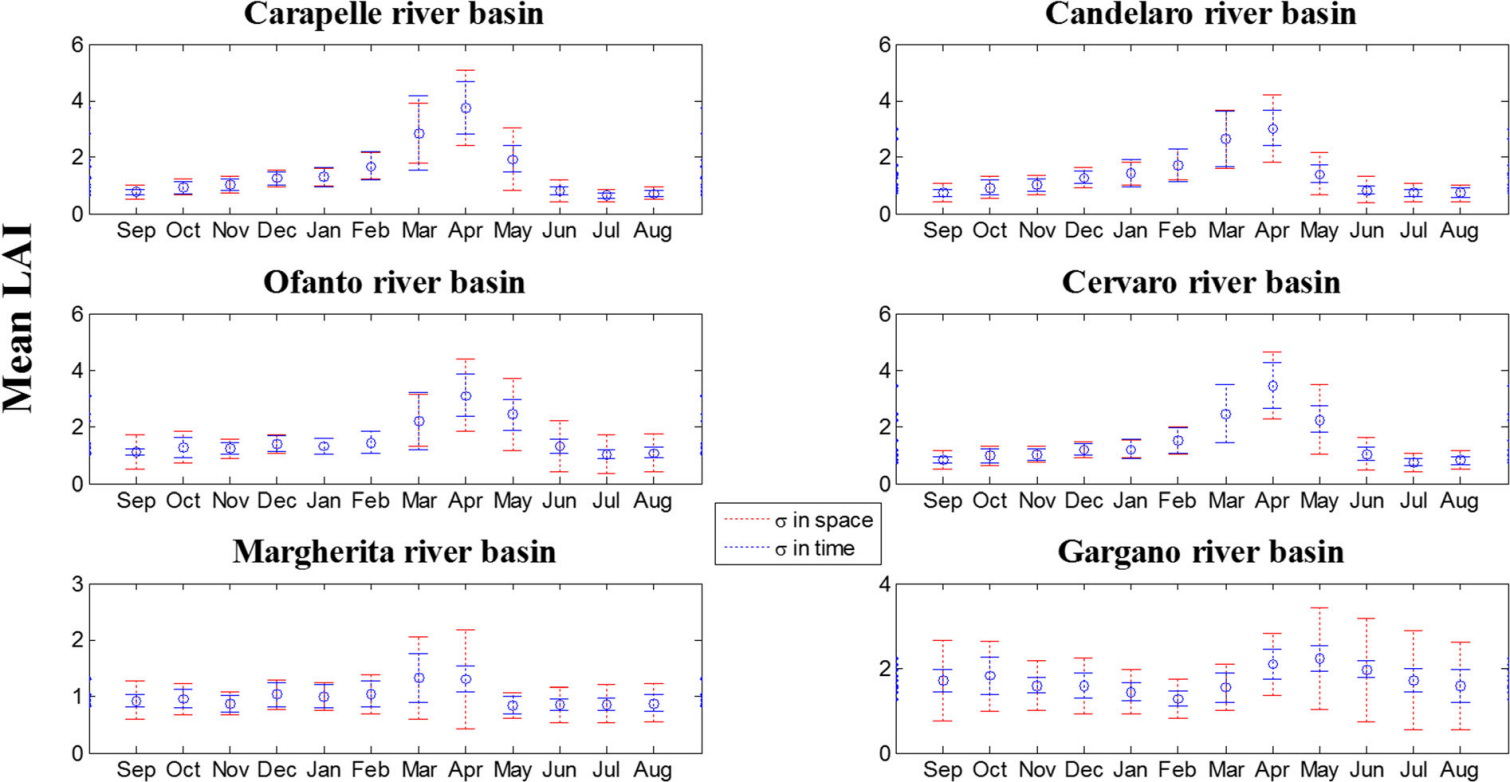
LAI monthly average in space and time ± standard deviation in space and standard deviation in time

The graphs in Fig. 3 show a comparison of space and time patterns. The blue circles indicate the monthly average in time and space. The blue dashed line represents the standard deviation in time, over the observed years, and the red dashed line represents the average standard deviation in space, across the basin area.

The subplots show the general trend of LAI in the hydrological year, characterised by a maximum value in April for Carapelle, Candelaro, Ofanto and Cervaro River Basins where the main production is wheat, in March for Margherita River Basin where fruit trees dominate the area and in May for Gargano River Basin where natural vegetation is the most widespread feature; the LAI shows its minimum values in July and August for Carapelle, Candelaro, Ofanto and Cervaro River Basins, in May and June for Margherita River Basin and in January and February for Gargano River Basin.

It is interesting to note that the standard deviation in space is generally greater than the standard deviation in time denoting that the heterogeneity in land cover and local effects usually prevails on the inter-annual climatic forcing. This was regularly observed in the Gargano area where natural vegetation prevails over agricultural species. On the other hand in all other basins, where agricultural species dominate, less variability was observed (i.e. less biodiversity) and spatial variability was recorded as almost equal to (sometimes overtaken by) variability in time.

**Fig. 4:**
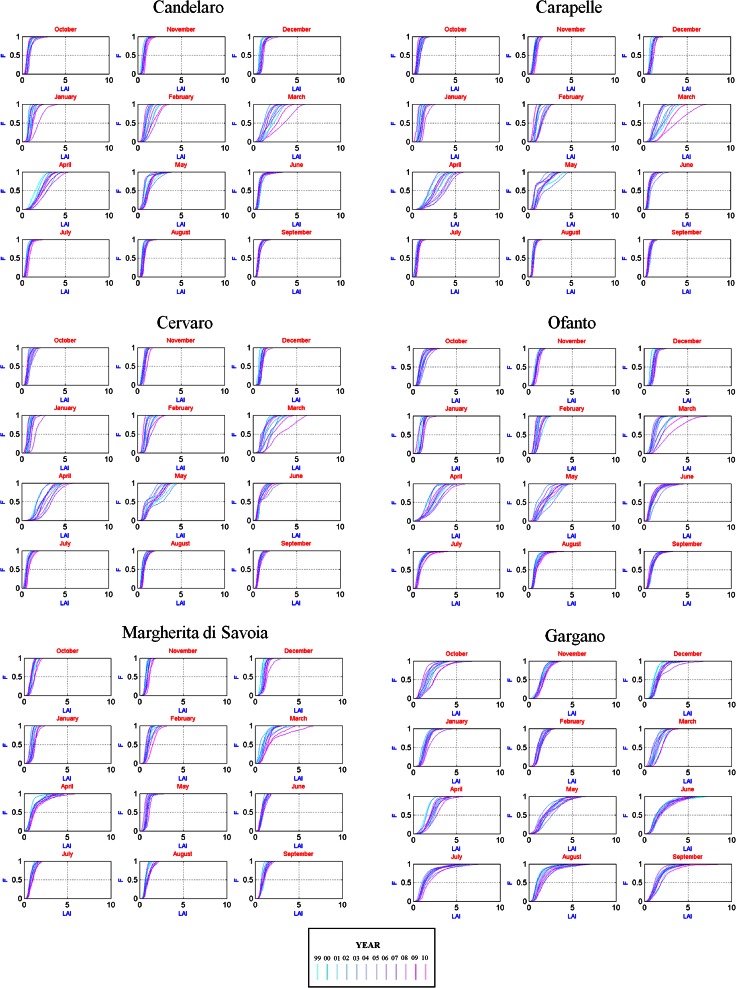
LAI cumulative frequency distributions (CFD) in space for each month of the year for each basin, over the 12 years

Figure 4 shows the LAI cumulative frequency distributions (CFD) for each basin and for each month of the year for all the years of observation. The distributions observed for the months of March and April are particularly scattered with a few years that reach rather high values (for example 2008 and 2010). This may depend on climatic factors such as rainfalls as well as anthropic factors but also from bias and noise in LAI retrieval.

As a positive feature of the method used for LAI estimation, which did not imply the use of calibration parameters, well differentiated distributions were obtained in the months of the wheat life-cycle and compact and almost homogeneous distributions in the other months.

Following these preliminary results, an extensive regression analysis was performed, considering the time series of annual LAI values, for all pixels (1 km^2^) included in the study area.

The method of linear regression analysis is widely used in vegetation dynamics detection with time series data. If time *t* is set as the independent variable with VI value of each pixel for dependent variable, the slope of linear regression is an effective index to quantify the trend of vegetation dynamics in the study period. In details, a positive value of the slope refers to a positive trend of vegetation dynamics, which means increasing of vegetation coverage or enhancing of vegetation activity, whereas a negative value of the slope refers to a negative trend of vegetation dynamics, which means decreasing of vegetation coverage or weakening of vegetation activity.

The goodness of fit of the linear regression is provided by the coefficient of determination *R*^2^ which is in this case equal to the square correlation coefficient (*r*) evaluated as

4 $$ r=\frac{cov\left(i,LA{I}_i\right)}{\sqrt{Var(i)Var\left(LA{I}_i\right)}} $$

where *i* is the serial number of the year in the study period, *LAI*_*i*_ is the LAI value in the year *i*, *cov* indicates the covariance and *Var* is the variance.

Moreover, a significance test of vegetation dynamics trend was conducted using the *p* value statistic with a significance level of 0.05.

**Fig. 5:**
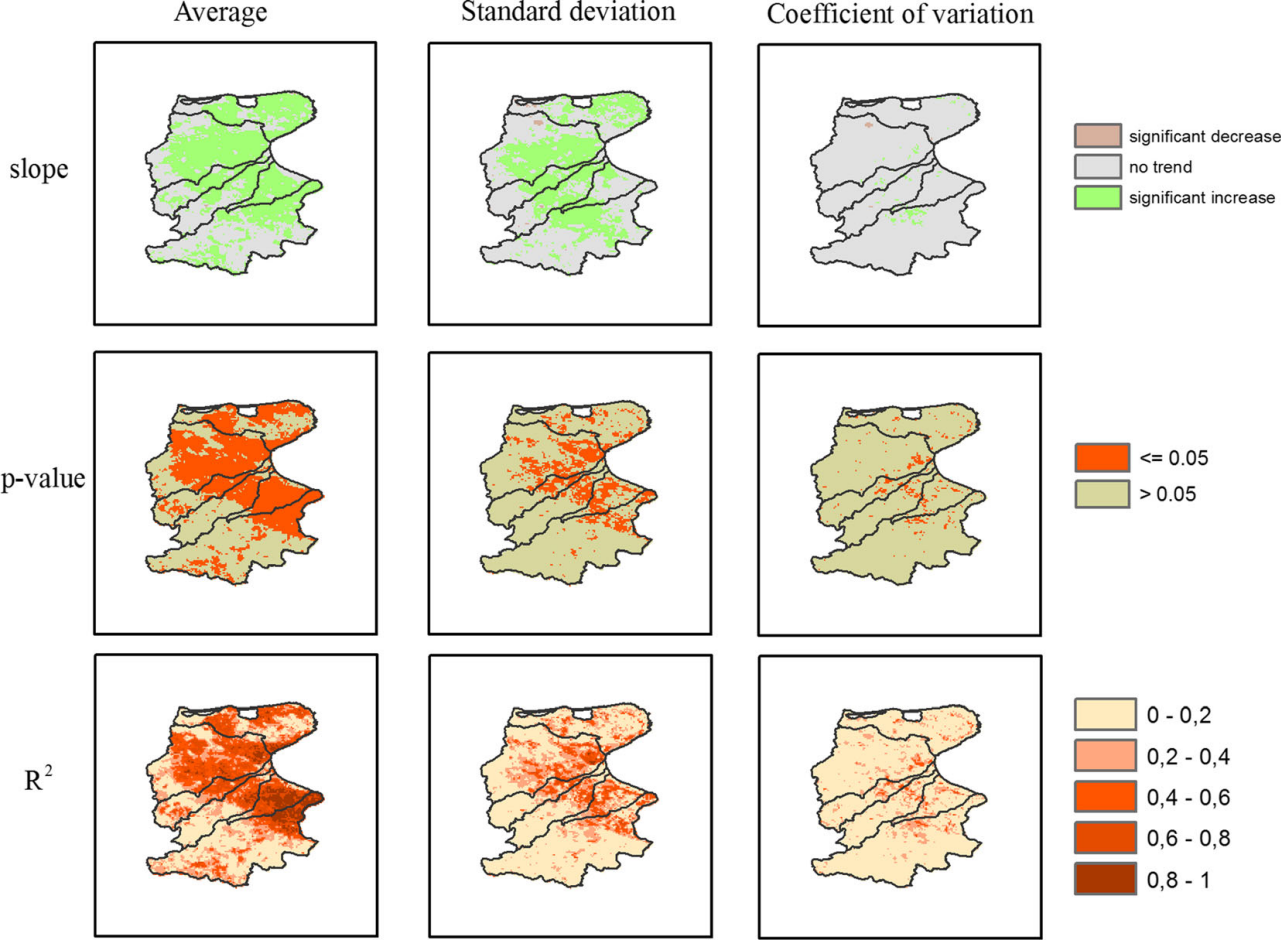
LAI linear trends observed over the 12 years studied, obtained for each pixel annually

Figure 5 represents the linear trends observed over the 12-year row studied, obtained for each pixel in terms of LAI annual average, standard deviation and coefficient of variation (which is the ratio between the standard deviation and the mean). Both the average and the standard deviation show a significant positive trend in large areas, as confirmed by the maps of *p* value and *R*^2^, especially in the valleys of the river basins studied and in the Tavoliere plain. On the other hand, the coefficient of variation does not show a similar increase, apart from a reduced number of pixels. This implies that the great majority of pixels have an increase in both mean and standard deviation values of the annual distribution of LAI.

A remarkable percentage of the study area shows a positive trend (about 54 %), which is widely and almost homogeneously observed in the Tavoliere plain with maximum values of 75 % in the reclamation ground of Margherita di S., followed by the Gargano and Candelaro areas with 66 % (see Table 1).

**Table 1:** Land use distribution in the study area and in areas (A+) with positive trend of annual average LAI

Study area		Gargano		Candelaro		Cervaro		Carapelle		Margherita di S.		Ofanto	
A [km^2^]	A+ [%]	A [km^2^]	A+ [%]	A [km^2^]	A+ [%]	A [km^2^]	A+ [%]	A [km^2^]	A+ [%]	A [km^2^]	A+ [%]	A [km^2^]	A+ [%]
8951	54.2	1427	65.9	2331	65.5	787	46.4	987	45.2	616	74.9	2803	39.6
Land use		% of A	% of A+	% of A	% of A+	% of A	% of A+	% of A	% of A+	% of A	% of A+	% of A	% of A+
Natural vegetation (woods)		63.5	72.3	10.6	8.2	18.0	12.9	9.4	3.4	0.5	0.2	20.8	18.9
Wheat and other cereals		10.4	7.3	61.1	65.1	63.8	67.5	73.4	73.5	33.4	34.0	51.8	43.2
Fruit trees (olive, grape, citrus, etc.)		11.6	14.3	12.4	12.6	5.3	7.7	6.6	9.3	40.0	48.2	13.2	24.8
Vegetables		7.2	0.8	9.4	9.4	7.6	8.4	7.7	11.7	14.2	14.7	8.5	8.0
Other vegetated areas		5.7	4.7	4.5	3.3	2.8	2.2	1.9	1.6	9.6	1.7	4.0	4.0
Urban areas		1.6	0.5	2.1	1.5	2.4	1.4	1.1	0.6	2.3	1.1	1.7	1.2

The effect of land use on the increase of LAI was analysed based on the land-use map of S.I.G.R.I.A. (INEA 1999). To the purpose of this analysis, we used a grid map with cells of 90 × 90 m. Gargano is the only area where natural vegetation (mainly forest) dominates the land cover map (63 %, see Table 1), while agricultural vegetation mainly characterises all the other basins. Wheat and other cereals are most widespread in Ofanto (51.8 %), Candelaro (61.1 %), Carapelle (73.4 %) and Cervaro (63.8 %) basins, whereas fruit trees (40 %) particularly dominate the area of Margherita di Savoia.

Table 1 also shows the areal land-use distribution for the six areas shown in Fig. 1 and the percentage of the same land-uses within the area (A+) with positive trend of the annual mean LAI. The percentages of land-use in the areas with positive trends shown in Table 1 substantially mirror the dominant distribution of land-use observed in the different areas; nevertheless, small differences between the percentage of land-use in the whole basin and the percentage of land-use in the area with positive trend were observed.

Based on the above observations, we focused our attention on the land covers that mainly explain positive trends, i.e. related to the Gargano area, shown in Fig. 5. As shown in Table 1, about 72 % of the area with observed positive trend of LAI is covered in forest within the Gargano area. In the Ofanto Basin, forest and wheat cover about 63 % of the area with positive trend. In the Candelaro, Carapelle and Cervaro Basins, wheat covers 65, 68 and 64 % of the area with positive trend, respectively.

These further analyses were carried out focusing on the inter-annual behaviour of the maximum spatial average LAI observed for each year for the different land uses, using the spatial average LAI observed in May for forest and April for wheat.

**Fig. 6:**
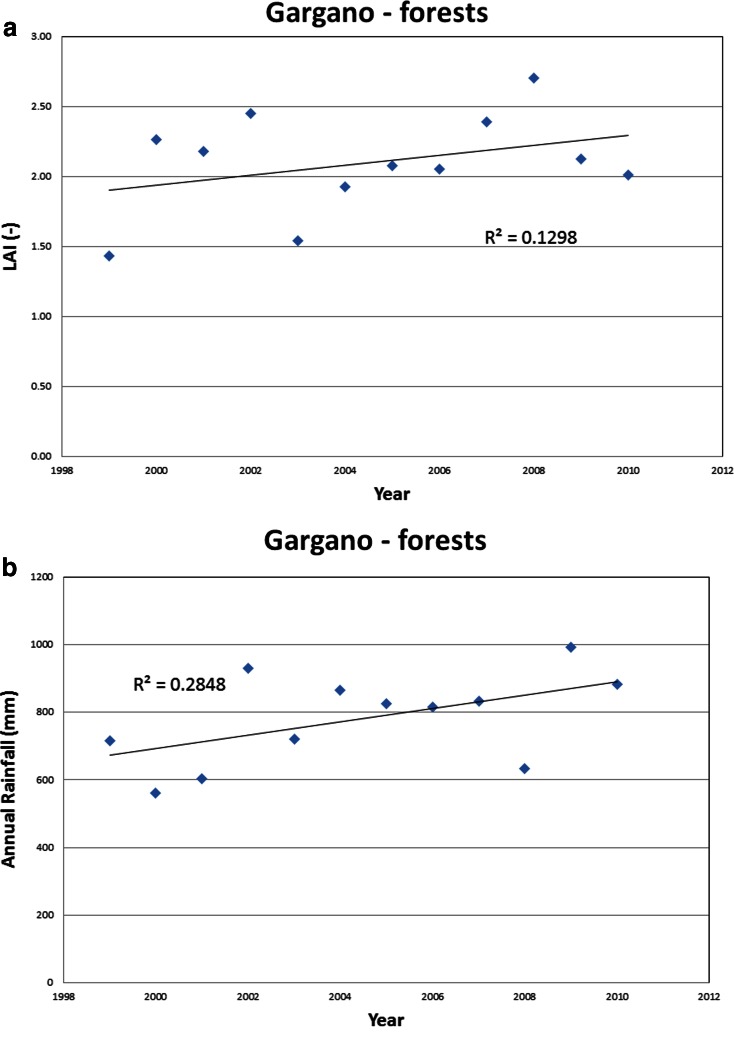
Observed time series of **a** spatial-average maximum LAI (observed in May) in the forest of the Gargano area and **b** total annual rainfall in the same area

A general increase was observed in the time series of spatial-average LAI (as in the case of the LAI of forest in the Gargano area shown in Fig. 6a) in terms of both natural and agricultural species, but this cannot be simply related to rainfall amounts. In fact, even considering that rainfall was characterised by weakly increasing annual amounts in the same 12-year row (1999–2010), as shown in Fig. 6b, against all expectations, no significant direct correlation was found between the maximum LAI and annual rainfall. Such result was also confirmed by evaluating the cross-correlation after detrending the maximum LAI and annual rainfall time series. No significant autocorrelation was found in both maximum LAI and annual rainfall time series either.

In literature, similar results have also been reported in other studies. Smettem et al. 2013 for instance observed that the forest leaf area index in South-Western Australia was not “tightly coupled to inter-annual variations in rainfall”. Yet, such result cannot be considered as an indicator of independence because a very low correlation coefficient may also result from the presence of a source of negative correlation, which would balance the positive one. Therefore, we performed further analysis in order to better assess the dependence of LAI on rainfall or other climatic variables and also to further explain the role of different land cover in the positive trend of LAI.

We performed several trial analyses in order to find the main climatic factors affecting LAI, including the analysis of the correlation of the spatial-average LAI in the month when the maximum value is reached with the observed monthly and annual data of rainfall and temperature.

**Fig. 7:**
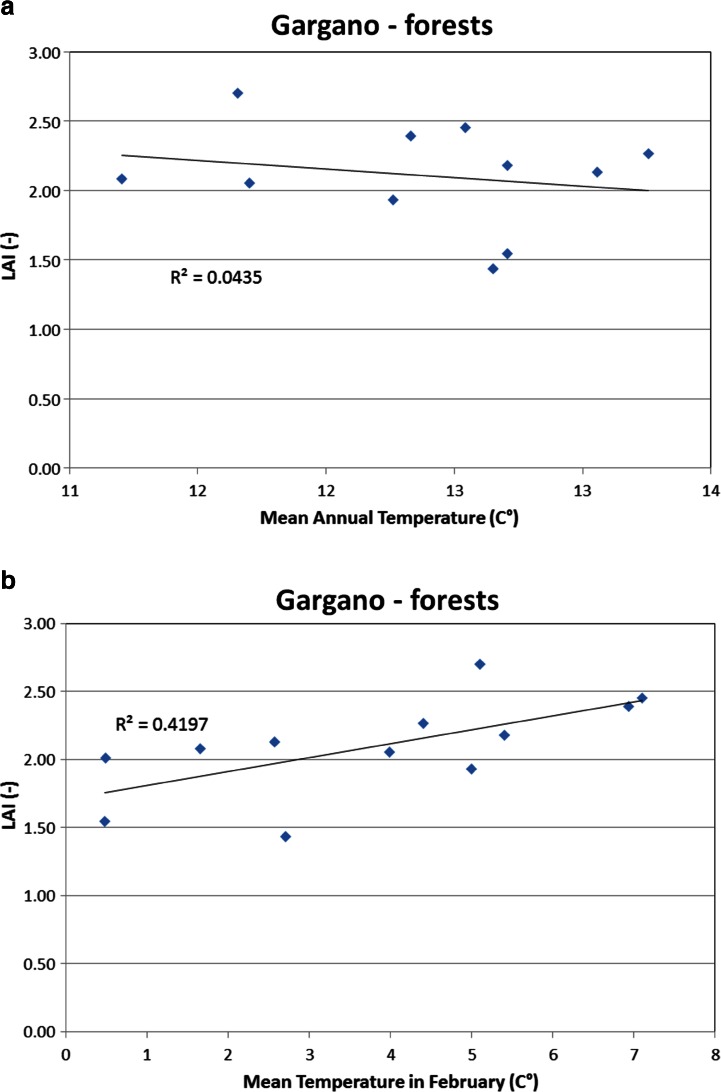
Linear correlation between spatial-average LAI in May in the forest of the Gargano area and mean temperature of February in the same area

We found that the spatial-average LAI behaviour in time was affected by temperature more than by rainfall. Interestingly, a significant correlation (see Table 2) was found between maximum annual LAI and temperature in February in Gargano’s forest and Ofanto’s wheat and forest (as shown in Fig. 7b) and between maximum annual LAI and temperature in January in the wheat area of Candelaro. The correlation with respect to mean annual temperature is always low (Fig. 7a). In fact, January and February are crucial months in the vegetative period because they often provide the minimum annual temperature.

**Table 2:** *R* ^2^ and *p* value of linear correlation between maximum annual LAI and temperature

Study area	Temperature			Rainfall		
	Month	*R* ^2^	*p* value	Month	*R* ^2^	*p* value
Gargano (forests)	February	0.3428	0.0455	January	0.2854	0.0736
Candelaro (wheat)	January	0.4547	0.0162	June	0.4056	0.0259
Cervaro (wheat)	February	0.2273	0.1171	January	0.1985	0.1466
Carapelle (wheat)	March	0.2644	0.0872	June	0.2548	0.0942
Ofanto (wheat)	February	0.4484	0.0172	January	0.4804	0.0124
Ofanto (forests)	February	0.7147	0.0005	January	0.3506	0.0425
Margherita di S. (fruit trees)	January	0.2152	0.1769	July	0.2993	0.0656

**Fig. 8:**
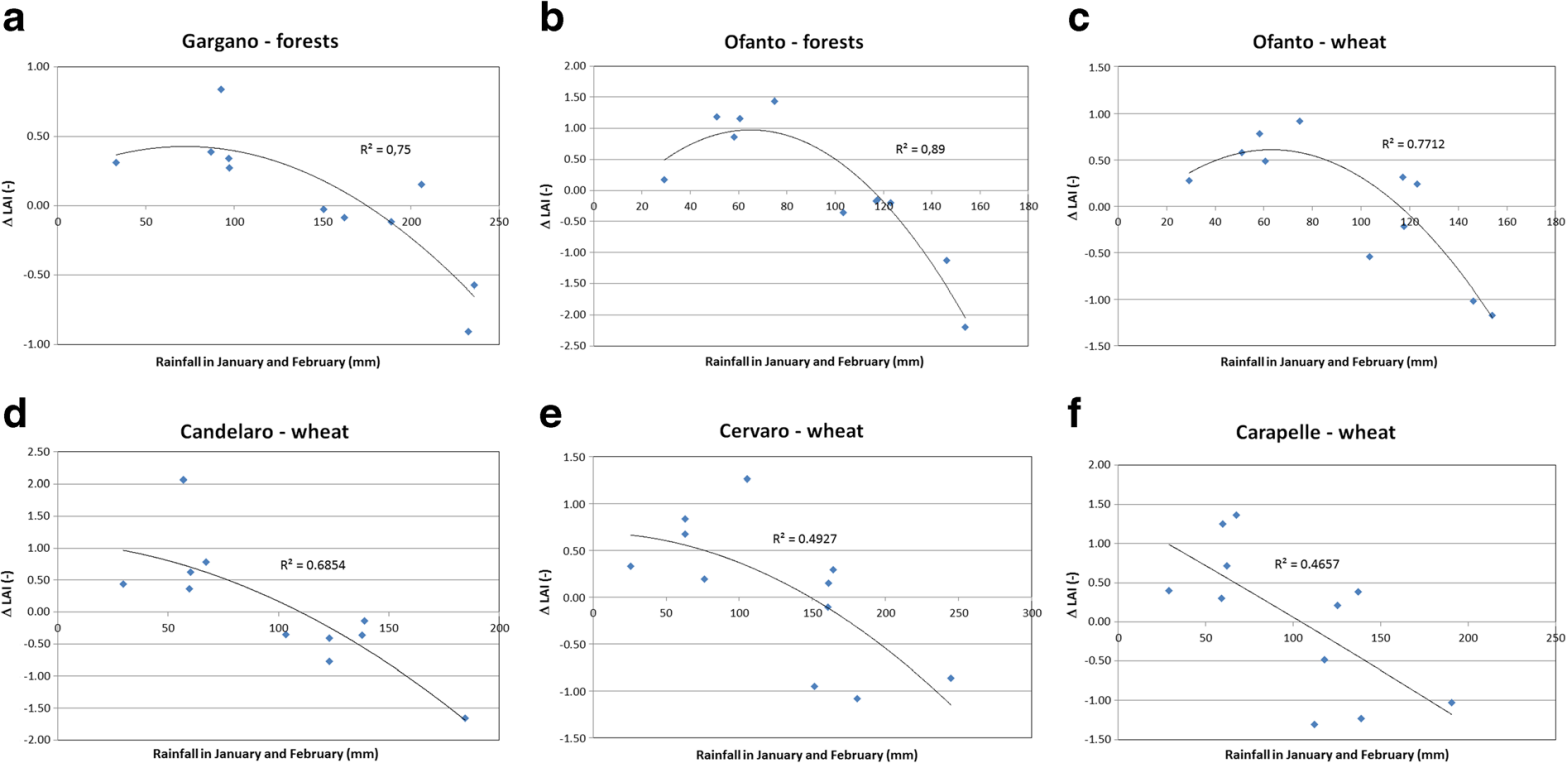
LAI patterns observed for forest (**a**–**c**) and wheat (**d**–**f**)

We also found an important influence of rainfall by considering the inter-annual increments of spatial-average LAI, ΔLAI, defined as the difference between the maximum value observed in any current year and the one observed in the precedent year. Figure 8 provides results of a simple regression analysis between ΔLAI and rainfall amounts in January and February of current years. Obviously, the rainfall amount in January and February is a significant part of the annual rainfall amount in the study area, and it may represent a per cent fraction ranging from 5 to 30 % or more of the total rainfall amount. Unlike for the annual rainfall amount, a further analysis of the time inter-annual pattern of the rainfall amount in January and February did not detect any significant trend. Although ΔLAI may be considered as a substantially detrended quantity, the quantity of ΔLAI also has an interesting physical meaning being it the measure of inter-annual vegetation turnover with reference to the maximum leaf area extent. Figure 8 shows a polynomial of second order which highlights that the relationship between LAI and rainfall is not always monotonous. The value of *R*^2^ ranges between 0.49 and 0.89. The relationship between rainfall and ΔLAI is different above and below the critical rainfall threshold of about 100 mm. In fact, ΔLAI shows a positive correlation with rainfall for rainfall values below or around the 100-mm threshold. When the rainfall amount exceeds such a threshold, the correlation turns to negative. This behaviour is commonly observed with both natural (forest, see Fig. 8a, b) and cultivated (wheat, see Fig. 8c–f) vegetation and is due to the fact that January and February are crucial months for the vegetative period because they are characterised by the lowest annual temperatures and by a significant fraction of the total annual rainfall amount.

## Conclusion

Using a VGT S10 data set related to the years going from 1999 to 2011, LAI variability was analysed in both space and time finding crucial insights for the description of the hydrologic dynamics characterising large parts of Puglia (Southern Italy). The high-quality temporal and spatial resolution of SPOT VGT sensor data provides significant contribution to land cover and vegetation monitoring both at global and regional scales.

LAI is an important state variable that can be used as input or control variable in distributed hydrological modelling. LAI distribution in space and time strongly affects important processes such as interception and evapotranspiration. LAI maps are also used as hydrological products in order to monitor vegetation stress and/or agricultural productivity.

Inter- and intra-annual variability depends on climatic variables (annual and monthly rainfall amounts), land cover and geomorphology, anthropic factors related to agronomic practices.

As a general behaviour of LAI in the area, over the decade studied, an increasing trend in annual average LAI was observed. This trend generally showed in the area independently from the land cover type (natural or agricultural). While a general increasing trend was also observed in rainfall annual amounts, the time series of spatial average LAI and annual rainfall did not show significant direct cross-correlation. This result is in line with the worldwide recognised complexity in explaining LAI variability which depends on several hydrologic and physiologic factors. Nevertheless, a number of interesting dependencies of LAI on climate factors were also found when analysing different species (forest and wheat) and focusing on the annual maximum spatial-average LAI, recorded in May for wheat and in April for forest. This supports the existence of a positive correlation between the annual maximum spatial-average LAI and the average temperature in the month of February (annual-minimum monthly temperature). A significant relationship between the annual maximum spatial-average LAI and the rainfall amount observed in January and February was also observed. In particular, the inter-annual increments of LAI strongly decrease in years with heavy rainfall in January and February, and such descending relationship begins when rainfall exceeds a threshold of around 100 mm. Below such threshold, the relationship between LAI and rainfall in January and February is generally ascending.

Such behaviour is observed in both natural and agricultural species and may be explained because high rainfall amounts obviously imply the persistence in these months of low pressure atmospheric conditions, persistent cloud cover and lower solar radiance on canopy cover, for it is well known in farming that the excess of rainfall in January and February produces negative effects on wheat production due to waterlogged areas and flood damages. On the other hand, we believe that many other physical and phenological interactions may actually affect the vegetation development and inter-annual turnover, which needs further investigation involving a more specific analysis of agronomic and ecological factors.

## References

[CR1] Aquilino, M.; Novelli, A.; Tarantino, E.; Iacobellis, V.; Gentile, F. In *Evaluating the potential of GeoEye data in retrieving LAI at watershed scale*, SPIE Remote Sensing, 2014; International Society for Optics and Photonics, 2014, pp. 92392B-92311.

[CR2] Alberti G, Boscutti F, Pirotti F, Bertacco C, De Simon G, Sigura M, Cazorzi F, Bonfanti P (2013). A LiDAR-based approach for a multi-purpose characterization of Alpine forests: an Italian case study. iForest-Biogeosciences and Forestry.

[CR3] Andersen J, Dybkjaer G, Jensen KH, Refsgaard J, Rasmussen K (2002). Use of remotely sensed precipitation and leaf area index in a distributed hydrological model. Journal of Hydrology.

[CR4] Atzberger C, Eilers PH (2011). A time series for monitoring vegetation activity and phenology at 10-daily time steps covering large parts of South America. International Journal of Digital Earth.

[CR5] Brown L, Chen JM, Leblanc SG, Cihlar J (2000). A shortwave infrared modification to the simple ratio for LAI retrieval in boreal forests: an image and model analysis. Remote Sensing of Environment.

[CR6] Brown ME, Pinzón JE, Didan K, Morisette JT, Tucker CJ (2006). Evaluation of the consistency of long-term NDVI time series derived from AVHRR, SPOT-Vegetation, SeaWiFS, MODIS, and LANDSAT ETM+ sensors. Geoscience and Remote Sensing, IEEE Transactions on.

[CR7] Capodici F, D’Urso G, Maltese A (2013). Investigating the relationship between X-Band SAR Data from COSMO-SkyMed Satellite and NDVI for LAI detection. Remote Sensing.

[CR8] Chen J, Jönsson P, Tamura M, Gu Z, Matsushita B, Eklundh L (2004). A simple method for reconstructing a high-quality NDVI time-series data set based on the Savitzky–Golay filter. Remote Sensing of Environment.

[CR9] Chen JM, Pavlic G, Brown L, Cihlar J, Leblanc S, White H (2002). Derivation and validation of Canada-wide coarse-resolution leaf area index maps using high-resolution satellite imagery and ground measurements. Remote Sensing of Environment.

[CR10] Chen P-Y, Fedosejevs G, Tiscareño-LóPez M, Arnold JG (2006). Assessment of MODIS-EVI, MODIS-NDVI and VEGETATION-NDVI composite data using agricultural measurements: an example at corn fields in western Mexico. Environmental Monitoring and Assessment.

[CR11] Dubovyk, O., Menz, G., & Khamzina, A. Trend analysis of MODIS time-series using different vegetation indices for monitoring of cropland degradation and abandonment in Central Asia. In *Geoscience and Remote Sensing Symposium (IGARSS), 2012 I.E. International, 2012* (pp. 6589-6592): IEEE.

[CR12] Ekercin S, Örmeci C (2010). Evaluating climate change effects on water and salt resources in Salt Lake, Turkey using multitemporal SPOT imagery. Environmental Monitoring and Assessment.

[CR13] Fassnacht KS, Gower ST, MacKenzie MD, Nordheim EV, Lillesand TM (1997). Estimating the leaf area index of north central Wisconsin forests using the LANDSAT Thematic Mapper. Remote Sensing of Environment.

[CR14] Gessner U, Niklaus M, Kuenzer C, Dech S (2013). Intercomparison of leaf area index. products for a gradient of sub-humid to arid environments in West Africa. Remote Sensing.

[CR15] Gigante V, Iacobellis V, Manfreda S, Milella P, Portoghese I (2009). Influences of leaf area index estimations on water balance modeling in a Mediterranean semi-arid basin. Natural Hazards and Earth System Science.

[CR16] Gutman G, Masek JG (2012). Long-term time series of the Earth’s land-surface observations from space. International Journal of Remote Sensing.

[CR17] Hagolle O, Lobo A, Maisongrande P, Cabot F, Duchemin B, De Pereyra A (2005). Quality assessment and improvement of temporally composited products of remotely sensed imagery by combination of VEGETATION 1 and 2 images. Remote Sensing of Environment.

[CR18] Houborg R, Boegh E (2008). Mapping leaf chlorophyll and leaf area index using inverse and forward canopy reflectance modeling and SPOT reflectance data. Remote Sensing of Environment.

[CR19] INEA, 1999.”S.I.G.R.I.A. Sistema informativo per la Gestione delle Risorse Idriche in Agricoltura”.

[CR20] Kidane Y, Stahlmann R, Beierkuhnlein C (2012). Vegetation dynamics, and land use and land cover change in the Bale Mountains, Ethiopia. Environmental Monitoring and Assessment.

[CR21] Kovacs J, King J, de Santiago FF, Flores-Verdugo F (2009). Evaluating the condition of a mangrove forest of the Mexican Pacific based on an estimated leaf area index mapping approach. Environmental Monitoring and Assessment.

[CR22] Lanorte A, Lasaponara R, Lovallo M, Telesca L (2014). Fisher–Shannon information plane analysis of SPOT/VEGETATION normalized difference vegetation index (NDVI) time series to characterize vegetation recovery after fire disturbance. International Journal of Applied Earth Observation and Geoinformation.

[CR23] Liu L, Jing X, Wang J, Zhao C (2009). Analysis of the changes of vegetation coverage of western Beijing mountainous areas using remote sensing and GIS. Environmental Monitoring and Assessment.

[CR24] Lovreglio, R., Leone, V., Giaquinto, P., & Notarnicola, A. (2010). Wildfire cause analysis: four case-studies in southern Italy. *iForest-Biogeosciences & Forestry, 3.*

[CR25] Maidment, D.R, (1992). Handbook of hydrology. McGRAW-HILL,INC, ISBN 0-07-039732-5.

[CR26] Maisongrande P, Duchemin B, Dedieu G (2004). VEGETATION/SPOT: an operational mission for the Earth monitoring; presentation of new standard products. International Journal of Remote Sensing.

[CR27] Martinez B, Cassiraga E, Camacho F, Garcia-Haro J (2010). Geostatistics for mapping leaf area index over a cropland landscape: efficiency sampling assessment. Remote Sensing.

[CR28] Miao L, Luan Y, Luo X, Liu Q, Moore JC, Nath R, He B, Zhu F, Cui X (2013). Analysis of the phenology in the mongolian plateau by inter-comparison of global vegetation datasets. Remote Sensing.

[CR29] Nemani RR, Running SW, Pielke RA, Chase TN (1996). Global vegetation cover changes from coarse resolution satellite data. Journal of Geophysical Research: Atmospheres (1984–2012).

[CR30] Nolè G, Murgante B, Calamita G, Lanorte A, Lasaponara R (2014). Evaluation of Urban Sprawl from space using open source technologies. Ecological Informatics.

[CR31] Passot, X. (2000). VEGETATION image processing methods in the CTIV. *Proceedings of VEGETATION*, *2*, 3-6.

[CR32] Peng J, Liu Z, Liu Y, Wu J, Han Y (2012). Trend analysis of vegetation dynamics in Qinghai–Tibet Plateau using hurst exponent. Ecological Indicators.

[CR33] Piragnolo M, Pirotti F, Guarnieri A, Vettore A, Salogni G (2014). Geo-spatial support for assessment of anthropic impact on biodiversity. ISPRS International Journal of Geo-Information.

[CR34] Portoghese I, Iacobellis V, Sivapalan M (2008). Analysis of soil and vegetation patterns in semi-arid Mediterranean landscapes by way of a conceptual water balance model. Hydrology and Earth System Sciences Discussions.

[CR35] Propastin P, Kappas M (2012). Retrieval of coarse-resolution leaf area index over the Republic of Kazakhstan using NOAA AVHRR satellite data and ground measurements. Remote Sensing.

[CR36] Qiu B, Zeng C, Tang Z, Chen C (2013). Characterizing spatiotemporal non-stationarity in vegetation dynamics in China using MODIS EVI dataset. Environmental Monitoring and Assessment.

[CR37] Rahman H, Dedieu G (1994). SMAC: a simplified method for the atmospheric correction of satellite measurements in the solar spectrum. Remote Sensing.

[CR38] Rinaldi M, Ruggieri S, Garofalo P, Vonella AV, Satalino G, Soldo P (2010). Leaf area index retrieval using high resolution remote sensing data. Italian Journal of Agronomy.

[CR39] Röder A, Udelhoven T, Hill J, Del Barrio G, Tsiourlis G (2008). Trend analysis of LANDSAT-TM and-ETM+ imagery to monitor grazing impact in a rangeland ecosystem in Northern Greece. Remote Sensing of Environment.

[CR40] Smettem KR, Waring RH, Callow JN, Wilson M, Mu Q (2013). Satellite‐derived estimates of forest leaf area index in southwest Western Australia are not tightly coupled to interannual variations in rainfall: implications for groundwater decline in a drying climate. Global Change Biology.

[CR41] Sonnenschein R, Kuemmerle T, Udelhoven T, Stellmes M, Hostert P (2011). Differences in LANDSAT-based trend analyses in drylands due to the choice of vegetation estimate. Remote Sensing of Environment.

[CR42] Stenberg P, Rautiainen M, Manninen T, Voipio P, Smolander H (2004). Reduced simple ratio better than NDVI for estimating LAI in Finnish pine and spruce stands. Silva Fennica.

[CR43] Telesca, L., & Lasaponara, R. (2005). Discriminating dynamical patterns in burned and unburned vegetational covers by using SPOT‐VGT NDVI data. *Geophysical Research Letters, 32*(21).

[CR44] Telesca L, Lasaponara R (2008). Investigating fire-induced behavioural trends in vegetation covers. Communications in Nonlinear Science and Numerical Simulation.

[CR45] Telesca L, Lasaponara R, Lanorte A (2008). Intra-annual dynamical persistent mechanisms in mediterranean ecosystems revealed SPOT-VEGETATION time series. Ecological Complexity.

[CR46] Tottrup C, Rasmussen MS (2004). Mapping long-term changes in savannah crop productivity in Senegal through trend analysis of time series of remote sensing data. Agriculture, Ecosystems & Environment.

[CR47] Vancutsem C, Pekel J-F, Evrard C, Malaisse F, Defourny P (2009). Mapping and characterizing the vegetation types of the Democratic Republic of Congo using SPOT VEGETATION time series. International Journal of Applied Earth Observation and Geoinformation.

[CR48] Verbesselt J, Somers B, Lhermitte S, Jonckheere I, Van Aardt J, Coppin P (2007). Monitoring herbaceous fuel moisture content with SPOT VEGETATION time-series for fire risk prediction in savanna ecosystems. Remote Sensing of Environment.

[CR49] Verbesselt J, Hyndman R, Newnham G, Culvenor D (2010). Detecting trend and seasonal changes in satellite image time series. Remote Sensing of Environment.

[CR50] Vermote EF, Tanré D, Deuze JL, Herman M, Morcette J-J (1997). Second simulation of the satellite signal in the solar spectrum, 6S: an overview. Geoscience and Remote Sensing, IEEE Transactions on.

[CR51] Yu, X., Zhang, A., Hou, X., Li, M., & Xia, Y. (2012). Multi-temporal remote sensing of land cover change and urban sprawl in the coastal city of Yantai, China. *International Journal of Digital Earth* (ahead-of-print), 1-18.

[CR52] Zheng G, Moskal LM (2009). Retrieving leaf area index (LAI) using remote sensing: theories, methods and sensors. Sensors.

